# Optimization and synergistic enhancement of microalgae productivity in laboratory raceway ponds via co-regulation of automated light-supplemented mixers and electric field system

**DOI:** 10.1186/s13068-025-02658-x

**Published:** 2025-06-14

**Authors:** Siyuan Ren, Cong Shao, Feifei Zhu, Michael Schagerl, Xinjuan Hu, Mostafa Sobhi, Ling Xu, Jingya Qian, Shuhao Huo

**Affiliations:** 1 School of Food and Biological Engineering, Jiangsu University, Zhenjiang, 212013 China; 2 School of Life Sciences, Jiangsu University, Zhenjiang, 212013 China; 3 Department of Functional and Evolutionary Ecology, University of Vienna, Djerassiplatz 1, 1030 Vienna, Austria; 4 Agricultural and Biosystems Engineering Department, Faculty of Agriculture, Alexandria University, Alexandria, Egypt; 5 Agricultural Engineering Department, Jiangsu University, Zhenjiang, 212013 China

**Keywords:** Microalgae, Raceway ponds, Computational fluid dynamics, Gas‒liquid mass transfer, Dynamic stirring, Electrostatic field

## Abstract

**Abstract:**

Raceway pond systems face inherent challenges in achieving optimal biomass productivity due to limitations in vertical mixing efficiency and uneven light distribution, compounded by the intrinsic dilute nature of phototrophic cultures. The combination of automated light-supplemented mixers and electric field treatment introduces a promising strategy to enhance raceway pond gas‒liquid mass transfer, improve microalgae biomass production, and increase carbon fixation. Computational fluid dynamics simulations identified an optimal mixing configuration employing a 75° inclined blade rotating counterclockwise at 300 rpm, which reduced dead zones from approximately 15.5% to 1.1% and shortened the light–dark exposure of cells to 2.7 s in a laboratory-scale raceway pond (71.4 dm^3^). Additionally, daily one-hour electrostatic field stimulation at 0.6 V cm⁻^1^ during the logarithmic growth phase significantly enhanced algal growth. The novel raceway pond system achieved a 20% increase in the productivity of *Limnospira fusiformis* and elevated the maximum carbon fixation rate to 0.14 g L⁻^1^ d⁻^1^, representing a 43% improvement and the high-value phycocyanin increased by 14.4%. This approach enhanced mixing efficiency and light utilization, providing a scalable strategy for high-value microalgae production in controlled bioreactors.

**Graphical abstract:**

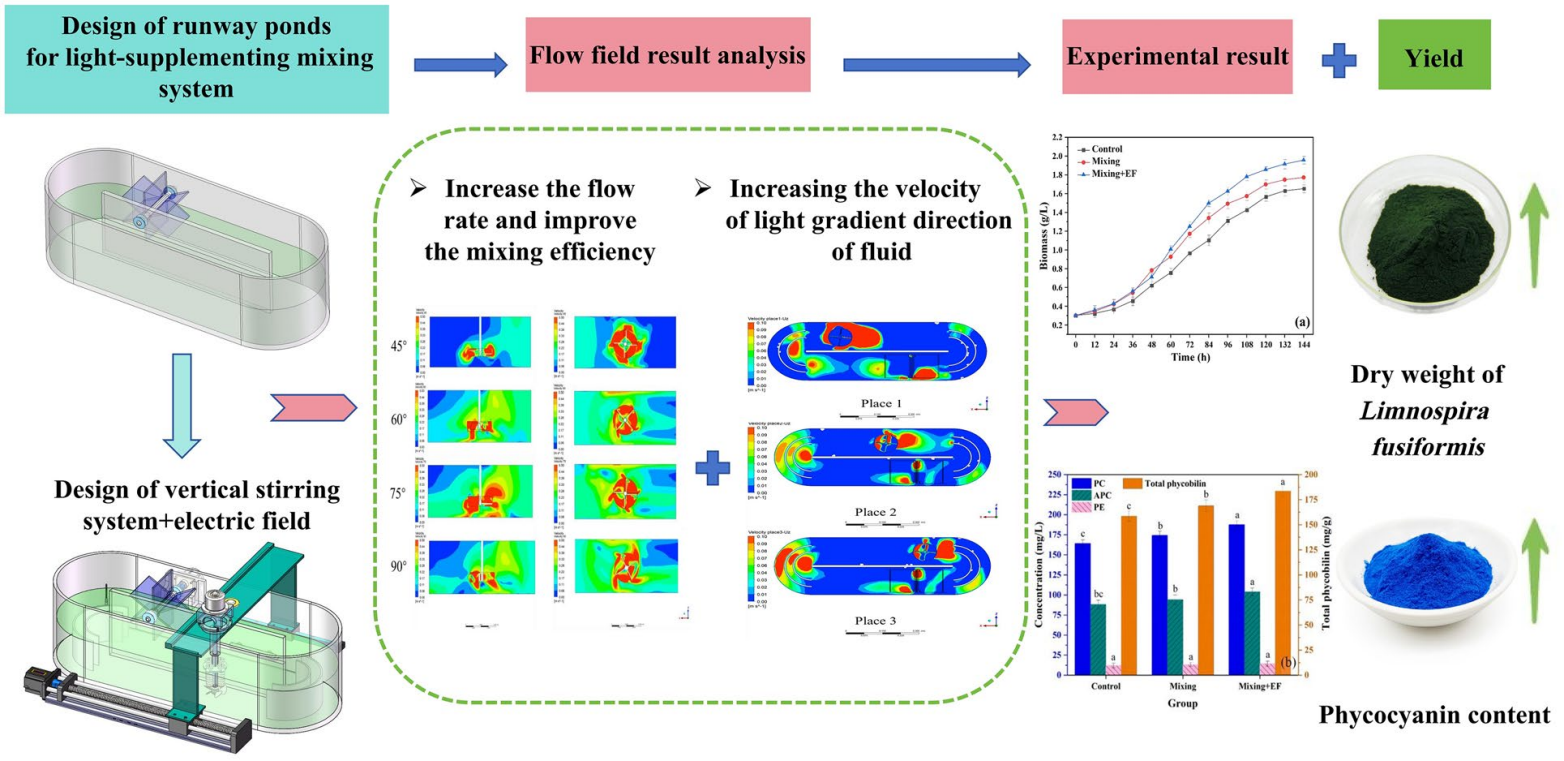

## Introduction

Microalgae represent a promising renewable resource with significant applications across various sectors, including nutrition, medicine, and animal feed [28]. Open raceway ponds (ORWPs) serve as the principal system for large-scale microalgae cultivation, accounting for 90% of global microalgae production [31]. Recent advancements have demonstrated the feasibility of large-scale microalgae cultivation for energy and bioproducts. For example, by optimizing the culture conditions and selecting promising strains of “Spirulina”, Zhu et al. [51] successfully realized the high-density cultures and converted them into biodiesel or biofuel, Shao et al. [35] discussed that the environmental benefits of microalgae culture can be maximized and its negative effects can be reduced by optimizing culture technology and management measures. Although ORWPs offer distinct advantages in terms of construction, cost, and operational use, they also present certain limitations [40]. Each cultivation method has advantages and disadvantages,there exists no cultivation method of choice. Photoautotrophic cultivation does not need media enriched in organic compounds, but photoautotrophic cultivation mostly results in lower biomass yield. Even with optimized setups, the dilute nature of phototrophic cultures will be kept, but within this general limitation, biomass generation can be optimized. Problems needed to be minimized include incomplete elimination of dead zones (> 15% volume in conventional designs [48]), light attenuation coefficients exceeding 2.5 m⁻^1^ at cell densities > 1.0 g L⁻^1^, severely limiting euphotic zone depth [42]. By synergistically optimizing hydrodynamics of the environment and cell physiology, these challenges can be overcome.

In recent years, increasing computer performance enabled significant advancements of computational fluid dynamics (CFD) simulations, which expanded the possibilities for improving the structural design of conventional ORWPs [52]. Numerous studies used CFD to analyze different ORWP systems. The hydrodynamic properties of raceway ponds are significantly influenced by their internal structure [33, 43], which affects how light and CO₂ are used by microalgae. Zhang et al. [49] demonstrated that adding a static mixer to ORWPs increased the vertical flow velocity and thus enhanced the mixing of algae, promoting microalgae growth. Cheng et al. [5] designed an up-down conversion baffle to increase light efficiency, reducing the light‒dark exposure of cells by 1/12 and increasing light efficiency by more than 30%. Legrand et al. [24] optimized the flow field of an annular ORWP using a motor impeller, reducing energy consumption and increasing the microalgae biomass yield. This method also minimizes dead zones and cell accumulation [21]. Akca et al. [1] demonstrated that vortex-induced vibration systems could enhance vertical mixing and light–dark cycling in raceway ponds, achieving a 12% biomass increase.

CFD remains vital in mixing system research, with numerical simulation results aligning well with experimental data [50]. Syrjänen and Manninen [39] simulated the flow field of a 45° inclined blade turbine by means of the Markov random field (MRF) method and k-ε and near-wall turbulence models, successfully replicating the wake vortex. Bakker et al. [2] applied the Sliding Grid (SG) method to calculate flow characteristics under different Reynolds coefficients in laminar flow areas and stated that this method yields more accurate results in the laminar flow region. Yianneskis [46] used the STAR-CD software to determine the distribution of turbulent kinetic energy dissipation in a stirred flow field with six straight blades, which turned out a basis for subsequent related researchers. Despite these advancements, critical gaps still remain: (1) Existing strategies often focus on singular optimizations (e.g., structural or hydrodynamic), neglecting metabolic reactions [1], (2Most studies fail to address the interplay between dynamic light regimes and cellular responses [5]; and (3) the integration of electric field boosting techniques with hydrodynamic optimization remains underexplored.

Electric field boosting techniques regulate bioprocesses primarily through targeted modulation of cell membrane permeability and metabolic activity. Appropriate electric field intensities induce reversible electroporation, enhancing transmembrane mass transfer efficiency while activating key enzymes like ATPases to accelerate energy metabolism cycles. This non-chemical intervention shows unique advantages in microalgae cultivation: low-intensity fields (< 100 V m⁻^1^) significantly enhance biomass and product synthesis by promoting CO_2_ nutrient uptake and lipid metabolism gene expression. For instance, Choi et al. [8] doubled both triacylglycerol content and biomass yield in Chlorella vulgaris using low-intensity fields, while Kumar et al. [22] achieved a 10% biomass increase in *Tribonema minus* during logarithmic growth via electrostatic stimulation. Shao et al. [36] reported that arc-shaped electrode deflectors improved “Spirulina” production capacity in raceway ponds. However, a single electric field stimulation remains insufficient to significantly reduce dead zones or enhance microalgae biomass production.

In this study, an additional mixing device and electric field treatment were combined to improve the productivity of microalgae from both the physical and biochemical perspectives. By optimizing the propeller device, the flow field and mixing efficiency in the open raceway pond were improved, creating a more suitable growth environment for microalgae, including shortening the light and dark exposure and reducing dead zones. Electric field treatment promote membrane permeability and metabolic activity via expression of genes related to photosynthesis. Combining the two approaches provides an optimization of the external physical environment and the regulation of cell physiology, thus minimizing the basic limitations of traditional ORWP. It is an innovative and comprehensive solution for more efficient cultivation of microalgae.

This study introduces two innovations: (1) CFD-guided vertical mixing with inclined blades in combination with pulsed electric field stimulation synchronized to the logarithmic growth phase. (2) Dynamic light supplementation synchronized with biomass concentration. We hypothesize that this integrated strategy will overcome the dual limitations of conventional ORWPs by simultaneously enhancing physical mass transfer and cellular metabolic activity. Through multiphysics simulations and controlled biological experiments, we quantify the synergistic effects on biomass yield, carbon fixation, and phycocyanin production, providing a scalable framework for high-value microalgae cultivation. Experiments were performed with *Limnospira fusiformis* (trade name Spirulina platensis) as a target organism, as this taxon is among the few ones that are already cultivated in large-scale ORWPs.

## Materials and methods

### Raceway pond system

**Fig. 1:**
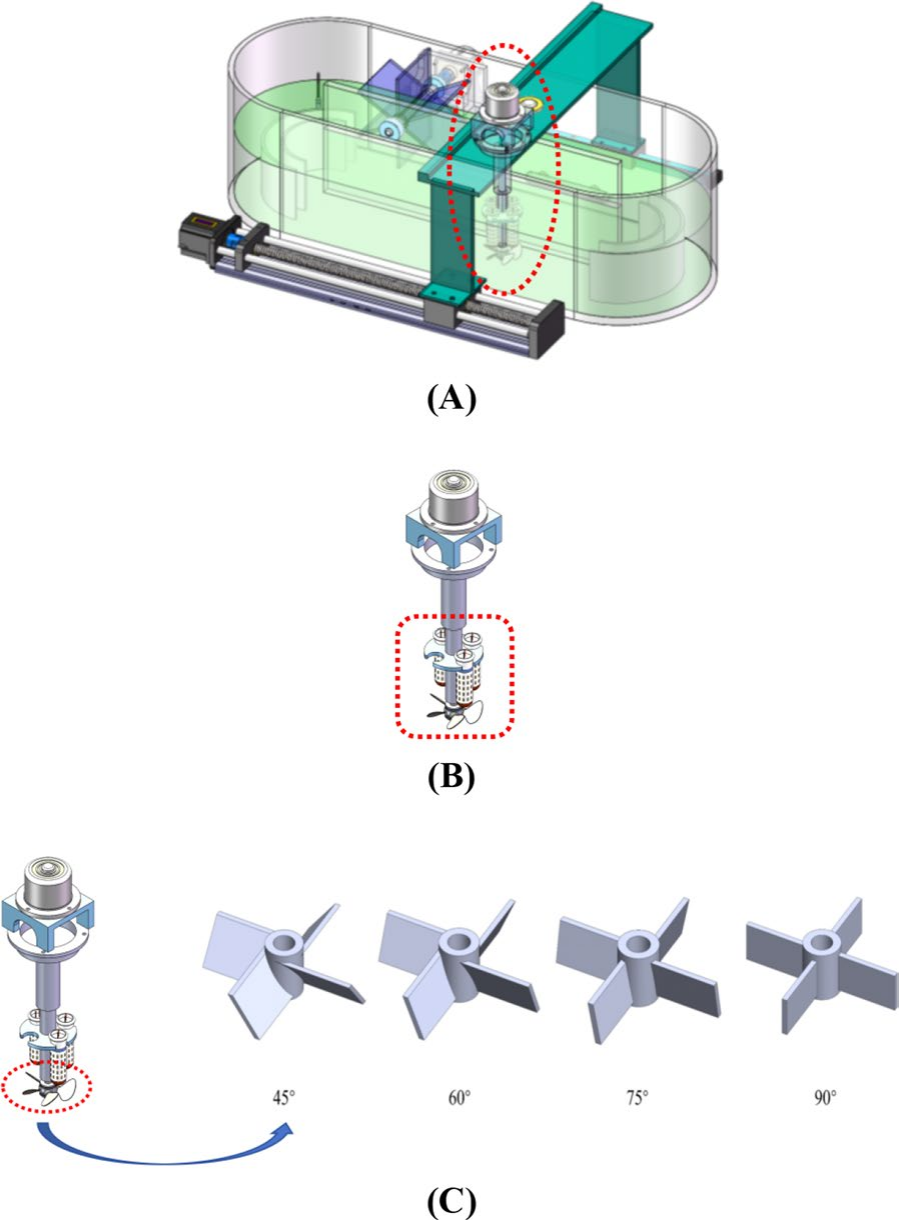
Overall design of the raceway pond. **A** Schematic diagram of the raceway pond structure. **B** Design of the automated light-supplemented mixers system. **C** Structural design of different blade angles

The experiments were conducted in a laboratory-scale ORWP with dimensions of 0.85 m (length) × 0.30 m (width) × 0.28 m (height). The culture medium level was 0.15 m, resulting in a constant volume 38.25 L, ensuring consistent hydrodynamic conditions across trials. The system incorporated arc-shaped electrode deflector structures (A-EDS) installed at the bottom curves (Fig. 1A). Each A-EDS consisted of inner and outer semicircular graphite electrodes (radii: 5 cm and 7.5 cm, respectively) spaced 2 cm apart, positioned 1 cm above the pond base to optimize flow guidance and electric field distribution. A paddlewheel (30 rpm) was installed for horizontal circulation, while a vertical stirring module (75° inclined blades, 300 rpm counterclockwise rotation) enhanced vertical mixing (Fig. 1B). A moving device was designed to enable the vertical stirring assembly to move horizontally along the length of the raceway pond. It consists of a main frame structure, a ball screw assembly, a connecting piece, and a guide rail slider component. The ball screw assembly is responsible for converting the rotational motion of the stepping motor into linear motion, allowing the stirring device to traverse the entire length of the raceway pond. The ball screw has a lead of 0.01 m and an effective stroke of 0.6 m, with a positioning accuracy of 0.002 m. The stepping motor used for driving the ball screw is a Pfizer 57BYG250B model, operating at 24 V with a rated current of 2.5 A and a holding torque of 1.2 N·m. The device is equipped with two limit sensors (HCYHTY SN04-N type) to ensure safe operation within the defined stroke range.

### Optimizing the blade structure of the mixing device

To enhance the mixing requirements in the ORWP, vertical mixing blades were modeled and designed with four straight blades selected (Fig. 1C). While ensuring that the total height of the blade structure was 2.5 cm, different designs were created for the blade inclination angles, including four blade structures with inclination angles of 45°, 60°, 75°, and 90°. The diameter of the blade structure's excircle was 8 cm, whereas the blade thickness was 0.2 cm. Additionally, there was an identical through-hole with a diameter of 0.8 cm in the center, allowing easy assembly with the transmission stirring shaft. To ensure cleanliness during production, polylactic acid (PLA), a biocompatible and microalgae-safe thermoplastic [34], was used for 3D printing the blades. The height of the blade structure was determined based on the dimensions and fluid dynamics requirements of the employed raceway pond. The blade height was set to 2.5 cm after considering the following factors: (1) hydrodynamic compatibility: The blade height was optimized to match the width of the raceway pond's long channel (0.15 m). This ensures that the blades can effectively disturb the fluid at the bottom of the pond, reducing dead zones and enhancing vertical mixing. (2) Microalgae growth requirements: The height of 2.5 cm was chosen to ensure that the blades do not obstruct the light gradient direction (Uz) while still providing sufficient vertical mixing force. This balance is crucial for maintaining optimal light exposure for microalgae growth. (3) Energy efficiency: A blade height of 2.5 cm was found to provide adequate mixing without excessive energy consumption. Higher blade heights would increase the required torque and power, while lower heights might not provide sufficient mixing efficiency.

### Automatic light-supplemented device

Light intensity is the most critical factor influencing microalgae production, as sufficient light is essential for optimized photosynthetic activity. Therefore, it is necessary to install a light sensor to monitor changes in light intensity, enabling real-time adjustments. In this system, a high-precision illuminance transmitter from Jianda Renke (Beijing, China) is selected for light intensity detection. This sensor is suitable for complex and variable production environments, with a physical layer utilizing RS485 bus for data transmission. It consumes less than 0.4 W of power and operates effectively within a temperature range of − 40 °C to 80 °C. The light intensity was maintained at 180 ± 5 μmol m⁻^2^ s⁻^1^ throughout the experiments, ensuring optimal growth conditions for *Limnospira fusiformis* [21]*.*

The light-supplemented device primarily consists of a slot and a light source. Its main function is to provide additional illumination during the production process when external light is insufficient or the algal concentration is too high, ensuring that production demands are met. The slot is a uniquely designed four-channel structure made of transparent acrylic, mounted on the stirring shaft sleeve, allowing it to move horizontally along with the stirring mechanism. The light source employs four 24 V high-brightness waterproof LED lights, each with a power of 12 W, providing a total power of 48 W. These LED lights are installed in the four channels of the slot and can be individually controlled to ensure uniform light distribution across the raceway pond.

### Temperature sensor and heater

To maintain optimal growth conditions for *Limnospira fusiformis*, a temperature monitoring and control system was integrated into the raceway pond system. A high-precision PT100 temperature sensor from Xingning Instrument (Shanghai, China) was used to continuously monitor the temperature of the culture medium. The sensor has a measurement range of 0 °C to 500 °C and an accuracy of ± 0.2 °C, ensuring precise temperature data collection.

The low-voltage DC heating rod from Mingxiang Electric Heating Technology (Yancheng, China) was employed to regulate the temperature. The heater is controlled by a relay, which activates the heating rod when the temperature drops below 27 °C, thereby maintaining the culture temperature within the desired range of 27 °C to 29 °C. This temperature control system ensures that the microalgae grow in a stable thermal environment, which is crucial for their growth and metabolic activities.

### CFD simulation analysis

**Fig. 2:**
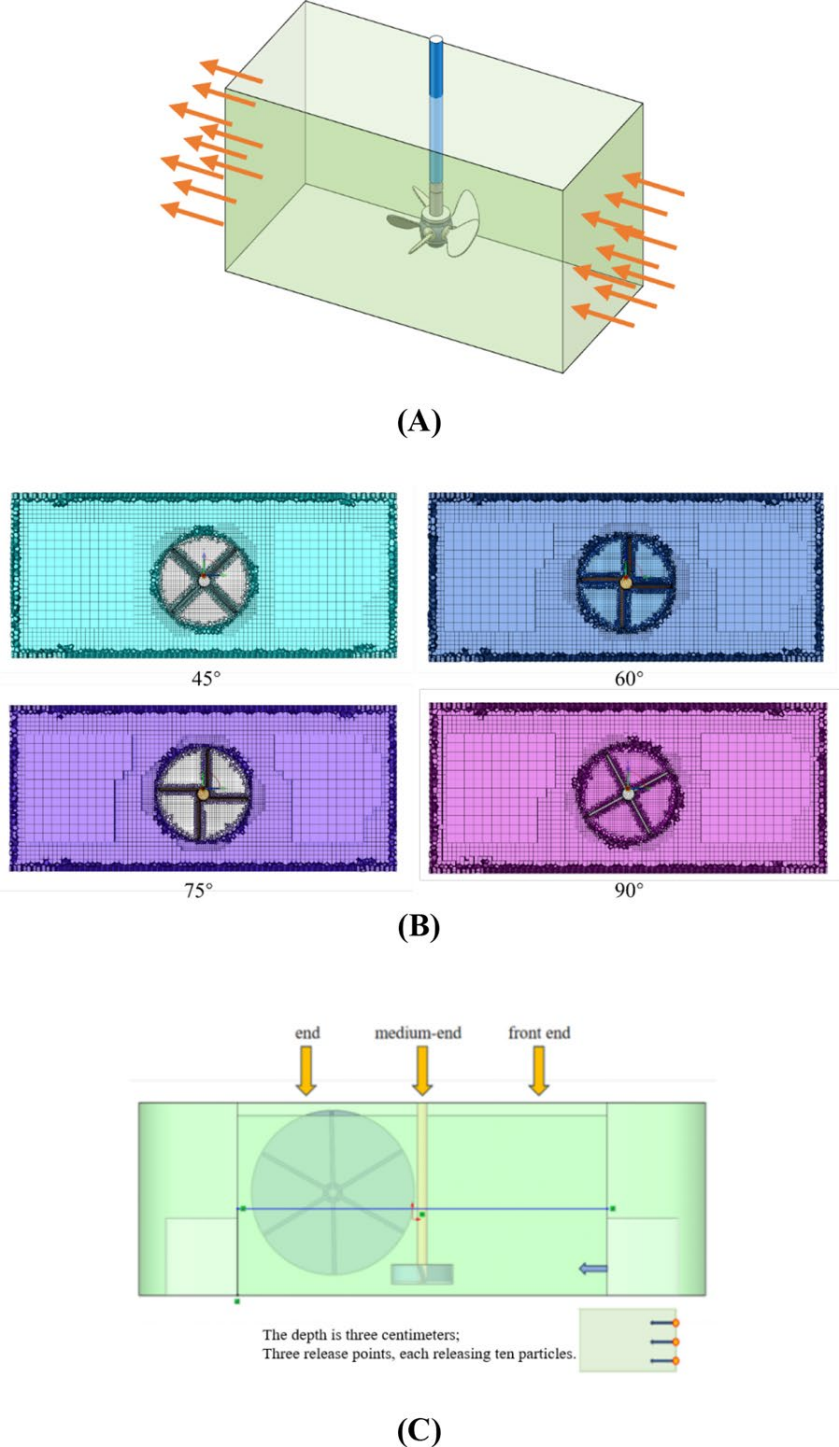
Fluid calculation area. **A** CFD calculation area. **B** Local fluid grid division of raceway ponds with blades and different angles. **C** Particle release point distribution

To determine the influence of different blade structures on mixing characteristics, the local mixing area with the blades in the long channel of the ORWP system was selected within the boundary of the simulation (Fig. 2A). The computational domain for the CFD simulation was a regular cuboid area measuring 0.35 m × 0.15 m × 0.15 m.

#### Computational methods

CFD simulations were performed using ANSYS Fluent 2022 R1. The eulerian multiphase model was employed to simulate the gas–liquid-solid (microalgae) interactions, treating the algal suspension as a continuous phase with dispersed CO₂ bubbles. Blade rotation was modeled via the Multiple Reference Frame (MRF) method, with transient simulations conducted using a pressure-based solver. The realizable k-ε turbulence model with enhanced wall treatment was selected for its accuracy in predicting rotational flows.

#### Mesh generation and validation

Mesh generation was coupled with a polyhedron-hexahedron mesh. To ensure stability and accuracy, the mesh in the blade rotation area was more refined than that in the fluid area farther from the rotation zone, with a mesh quality greater than 0.8. Mesh independence was confirmed via a Grid Convergence Index (GCI) < 3% for velocity magnitude at key monitoring points, validated against experimental Particle Image Velocimetry (PIV) data (RMSE < 0.15 m s⁻^1^).

#### Boundary conditions

Upper and left walls: pressure outlets, right wall: velocity inlet (0.18 m s⁻^1^) with turbulent intensity 5%, other walls: static no-slip walls, blades: rotating wall (300 rpm, counterclockwise).

#### Particle tracking analysis

To evaluate the performance of the ORWP, geometric models of the mixing device were selected at the front (Place 1), middle (Place 2), and end (Place 3) of the channel (Fig. 2C). Approximately 10 particles were released from each point and tracked iteratively for 100 s.The polyhedron–hexahedron grid divides the dynamic and static regions, which are coupled via the sliding grid method. The main impeller speed was set to 30 rpm, while the stirring speed was optimized to 300 rpm. A short using a Lagrangian Discrete Phase Model (DPM). The simulations were performed using ANSYS Fluent 2021R1, employing a pressure-based solver with a transient solution. The solver utilized a time step of 0.002 s, a total of 1000 time steps, and a maximum of 40 iteration steps per time step. This configuration ensured stable and accurate convergence of the solution, capturing the dynamic behavior of particles in the turbulent flow field.

### Microalgae culture and experimental analysis methods

#### Microalgae culture

The tested strain was *Limnospira fusiformis*, provided by the College of Food and Biological Engineering, Jiangsu University. The strain was chosen for its commercial value, fast growth rate, and known responsiveness to electrostatic field stimulation. Zarrouk culture medium was used with an initial pH of 9.5. *Limnospira* was first cultured in a column reactor inside a light incubator at 28 ± 2 °C with a light intensity of 180 ± 5 µmol photons m⁻^2^ s⁻^1^ (measured via LI-250A light meter) and then transferred to a custom-made ORWP for continuous cultivation [21]. The initial inoculation amount was 0.3 g L^−1^, and the pH was maintained between 9.2 and 10.2 throughout the 144 h culture period. A low-voltage DC heating rod (36 V, 200 W) from Mingxiang Electric Heating Technology (Yancheng, China) was used to maintain the temperature between 27 and 29 °C. When the temperature dropped below 27 °C, the relay controlled the heater to start working to keep the temperature within the desired range. Light intensity and temperature were monitored throughout the experiments using Jianda Renke sensor and a high-precision PT100 temperature sensor.

#### Determining the mixing time (MT) and mass transfer coefficient (MTC)

Seven liters of distilled water were added to the ORWP, and the paddle wheel speed was set to 30 rpm [27]. In order to estimate the mixing time (MT), the NaCl solution (0.1 M) was injected at three representative positions: (1) upstream of the stirrer (Place 1), (2) adjacent to the A-EDS (Place 2), and (3) near the paddlewheel (Place 3) (Fig. 2C). Conductivity was measured at a fixed monitoring point 15 cm downstream from the paddlewheel axis. MT is calculated from when the conductivity rises markedly until it fluctuates within ± 5%. All trials were repeated with injections at each position to minimize locational bias [25].

For MTC calculations, an InPro5000i/120 Mettler Toledo dissolved CO_2_ sensor was used for online measurement. First, 99.9% nitrogen gas was introduced into the microalgae suspension, which was then ventilated for 30 min to remove the dissolved CO_2_ from the solution. A mixed gas containing 15.0% CO_2_ was subsequently introduced into the ORWP, and the solution temperature was maintained at 25 °C. The change in the dissolved CO_2_ concentration in the ORWP was measured for 60 min, with data recorded every 30 s. The dissolved CO_2_ concentration was then calculated on the basis of the recordings [17]. Both the MT and MTC measurements were repeated three times for accuracy.

#### ***Biomass measurements and CO***_***2***_*** fixation efficiency***

Five milliliters of culture mixture was collected in a preweight and predried vial and centrifuged for 10 min at 5000 rpm via a high-speed centrifuge (DL-5-B, China). The sample was washed repeatedly with distilled water, centrifuged three times, and then dried at 60 °C until a constant weight was achieved. The dry mass was then calculated by subtracting the weight of the empty vial from the vial containing the dried material and multiplying by × 200 (recalculation to 1 L). The growth rate, which refer to the overall growth rate, respectively, were calculated via the following formulas provided by Brennan and Owende [4]:

1 $$Px=(X2-X1)/(t2-t1)$$

2 $$\mu =(ln X2-ln X1)/(t2-t1)$$

where X2 is the dry mass (g L^−1^) of *Limnospira* at time t2 (d) in the culture cycle and X1 is the dry mass (g L^−1^) at time t1 (d). t2 and t1 are the time intervals (d); Px represents the growth rate [g L^−1^ d^−1^].

The cellular carbon content (Xcbm) of the cells was detected via a CHN element analyzer (Vario Macro Cube). The carbon fixation rate, or CO_2_ fixation rate, was calculated via the following formula Chiu et al. [7]:

3 $$Rc=Px\cdot Xcbm\cdot {M}_{{CO}_{2}}/Mc$$

Rc represents the fixed rate of CO_2_ [g L^−1^ d^−1^]; Px represents the growth rate [g L^−1^ d^−1^]; Xcbm represents the carbon content (%) filaments; M_CO2_ represents the relative molecular mass of CO_2_; and Mc represents the relative molecular mass of carbon.

#### Phycobilins content

Phycobilin analysis followed Bennett et al. [3]. First, 0.5 g of dried microalgae biomass was placed in a container containing 0.1 mol L^−1^ phosphate buffer (pH 7.0). The mixture was then fully and evenly stirred with a stirrer, followed by ultrasonic treatment for 5 min. After ultrasonic treatment, the mixture was transferred to a volumetric flask and adjusted to a total volume of 250 ml with this buffer. Next, the 250 ml mixture was poured into a 300 ml plastic vial and frozen at 20 °C for 12 h. After that, the frozen mixture was thawed at 25 degrees Celsius. After thawing, a centrifuge (model DL-5-B) was used to centrifuge the mixture at a centrifugal force of 3000 rpm for 15 min. After centrifugation, the supernatant was collected. This freeze‒thaw process was repeated three times to ensure that the components in the microalgae biomass were fully released into the supernatant. After each freeze‒thaw cycle, the supernatant was collected. Finally, all the collected supernatants were combined, and their absorbance values were measured at three wavelengths: 620 nm, 652 nm and 562 nm. The phycobilin content was calculated via the following formula:

4 $$PC=({A}_{620}-0.474{A}_{652})/5.3$$

5 $$APC=({A}_{652}-0.208{A}_{620})/5.09$$

6 $$PE = [A_{562} - 2.14\left( {PC} \right) - 0.849\left( {APC} \right)]/9.62$$

7 $$Total\ phycobilin (mg\bullet {g}^{-1})=[(PC)+(APC)+(PE)]\times Vm$$

Phycocyanin (PC), Allophycocyanin (APC) and Phycoerythrin (PE) are the contents of phycocyanin, allophycocyanin and phycoerythrin in the supernatant (mg·ml^−1^), V is the volume of the supernatant (ml), and m is the sample dry mass (g).

#### Photosynthetic performance

Photosynthetic activity reflects the ability and efficiency of microalgae to convert light energy into chemical energy through photosynthesis, which significantly impacts microalgae growth and the carbon cycle [10]. We used a hand-held device for measuring the quantum yield (ΦPSII) and maximum photosynthetic efficiency (Fv/Fm): 5 mL of culture was collected and first allowed to acclimate in the experimental environment with an exactly defined irradiance of 200 μmol m⁻^2^ s⁻^1^ for 20 min. Then, the automated measuring routine for ΦPSII was started with actual fluorescence (Fs) measured first, followed by a saturating pulse for measuring maximum fluorescence under ambient light (Fm′). Calculation of ΦPSII$:$

8 $$\phi PSII=(Fm{\prime}-Fs)/Fm{\prime}$$

Afterwards, the sample was dark-adapted for 20 min to guarantee full relaxation of the photosystems. The minimum fluorescence F_0_ in the dark was measured, followed by the application of a strong saturating pulse to obtain the maximum fluorescence (Fm).

9 $$Fv/Fm=(Fm-Fo)/Fm$$

### Verification conditions for production experiments

In this production experiment, *Limnospira* was cultivated outdoors in a greenhouse via the designed dynamic mixing system of the ORWP. Three treatments were compared: (1) only A-EDS installed at the bottom of the raceway pond (control), (2) A-EDS combined with the optimized stirring strategy (mixing) and (3) the combined treatment (mixing + EF). *Limnospira* was cultivated to evaluate the production performance index of the reactor equipment. The EF treatment involved stimulation for 1 h per day during the logarithmic growth period (36 h, 60 h, 84 h, 108 h, 132 h) with a static electric field intensity of 0.6 V·cm^−1^ [22]. Samples were collected every 12 h to analyze biomass, pH, pigments and photosynthetic performance. The average value is obtained from running the system three times.

For all the experiments, the paddle wheel speed and main impeller speed of the raceway pond were set to 30 rpm, the stirring speed was set to 300 rpm, and the moving speed of the ball screw assembly was set to 0.02 m s^−1^. The stirring system and the ball screw assembly operated simultaneously and stopped together, with a 3 h rest period after every 5 min of operation. The depth of the culture medium in the ORWP was 15 cm, the initial inoculation amount was 0.3 g L^−1^, and the culture cycle lasted 144 h.

### Statistical analysis

OriginPro2024 and SPSS software were used for statistical analysis of the experimental data. All the data were analyzed via a completely randomized one-way ANOVA, and each experiment was repeated at least 3 times to ensure the accuracy of the data, with a significant difference level of P < 0.05.

## Results and discussion

### CFD simulation of the mixing area in vertical stirring system

**Fig. 3:**
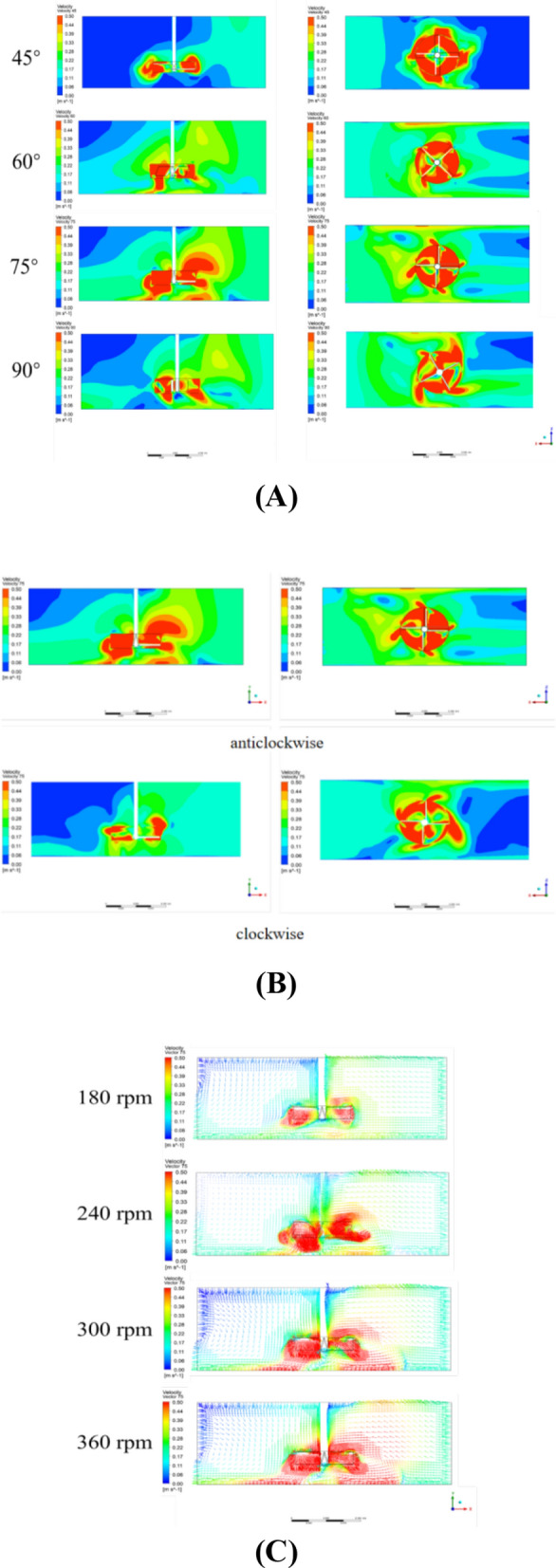
Schematic diagram of CFD numerical simulation results of the flow rate of the rotating pond under different operating conditions of the automatic light-filling mixer **A** Inclination angle **B** Operating direction **C** Rotation speed

The simulation results revealed that during agitation, a high-speed zone formed in the rotating region, enhancing fluid motion in the direction of rotation. In Fig. 3A the 75° blade angle significantly improved the radial flow velocity, reducing fluid retention at the bottom. Notably, the 75° blade angle significantly improved the vertical flow velocity, which reduced dead zones from 15.5 to 1.1%. This result suggests that optimizing blade angle and direction plays a crucial role in improving fluid dynamics, which was also observed in similar CFD-based studies [21, 24]. According to Prakash et al. [32], this increased shear force enhances the mixing efficiency and reduces dead zones, improving mass transfer within the system. However, the impact of agitation on long-term microalgae growth remains less studied, and this work aims to bridge this gap by providing experimental evidence. With a 45° blade angle, the vertical velocity of the medium net to the blade and the incident flow velocity at the horizontal plane did not significantly improve because of inadequate disturbance of the bottom fluid. This was likely caused by the 45° inclination of the rotor blades, resulting in low mixing efficiency and insufficient rotational thrust. The incident fluid and swirling flow induced by rotation may have reached a balance, limiting the overall velocity increase within the long channel.

As the blade angle increased to 60°, the vertical flow velocity notably increased, with a maximum velocity of 0.41 m s^−1^ at the blade bottom interface. A further increase in the blade angle to 75° significantly elevated the radial velocity at the bottom of the pond, with the maximum cross-sectional radial velocity exceeding 0.5 m s^−1^. A 90° blade failed to further enhance mixing despite generating a large velocity field around the blade in the horizontal direction. It poorly disturbed the bottom fluid, possibly because excessive stirring and shear forces caused uneven stress and the formation of large vortices. This hindered effective bottom fluid agitation and impacted the overall mixing performance [32]. On the basis of this analysis, a 75° blade angle structure is recommended. The 75° blade angle effectively promoted vertical mixing and impacted the pond bottom, increasing the shear force and agitation force and enhancing the mixing efficiency. The horizontal velocities also improved compared with those of the previous two blade settings. We decided to use a 75° blade angle for subsequent experiments.

The study also tested the effects of the rotation direction. Clockwise rotation of the agitator impeller weakened the horizontal flow velocity at the inlet, creating a large area of low-speed zones, and the outlet velocity was similarly reduced (Fig. 3B). Specifically, the horizontal flow velocity decreased from 0.2 to 0.05 m s^−1^. This finding can be explained by the combined effect of the blade's inclined direction and the incident fluid, where the fluid impact generated by the clockwise rotating blade canceled out the incident fluid, failing to improve fluid performance before and after the rotating region [37]. In contrast, the 75° blade rotating counterclockwise generated more forward fluid impact and aligned with the incident flow direction [18]. This enhanced the horizontal velocity before and after the rotation region from 0.1 to 0.3 m s^−1^. Counterclockwise rotation also yielded more pronounced vertical mixing, significantly improved the overall flow field velocity, and enhanced the disturbance and mixing capabilities.

CFD simulations were conducted at four different impeller rotational speeds: 180 rpm, 240 rpm, 300 rpm, and 360 rpm (Fig. 3C). As the speed increased from 180 to 300 rpm, the disturbance effect of the agitation impeller on the bottom fluid increased by 150%, with the maximum bottom velocity at the characteristic cross-section rising from 0.28 to 0.7 m s^−1^. This indicates a substantial improvement in mixing capability toward the surface, thereby increasing the light supply and reducing the time required to complete the vertical light‒dark cycle by 40%. The enhanced mixing efficiency and vertical flow velocity significantly improved the overall fluid dynamics in the raceway pond [30], as evidenced by the reduction in dead zones from 15.5 to 1.1%.

For both existing ORWP, and future constructions, vortex-induced mixing is a measure, which is easily to integrate to promote large-scale vertical movement of algal cells, increasing the light gradient velocity [9]. The selection of an optimal agitation speed must consider mixing efficiency, but also energy consumption and economic feasibility. At lower speeds (e.g., 180 rpm), the mixing efficiency is insufficient to guarantee optimum light and nutrient supply for maximum growth rates. As the speed increases to 300 rpm, the mixing efficiency improves significantly, enhancing light supply and reducing dead zones, which support biomass generation. However, further increasing the speed to 360 rpm results in diminishing returns. The improvement in mixing efficiency is less than 10%. This fact highlights the importance of selecting an agitation speed that maximizes productivity while minimizing energy costs. Additionally, the operational feasibility of high agitation speeds must be considered. High-speed agitation can lead to increased mechanical wear and tear on equipment, and higher maintenance costs. These factors collectively impact on the long-term sustainability and economic viability of the system. Therefore, an agitation speed of 300 rpm is recommended, as it provides a favorable balance between mixing efficiency and energy consumption, ensuring both high productivity and economic feasibility.

### CFD simulation in the whole ORWPs

**Fig. 4:**
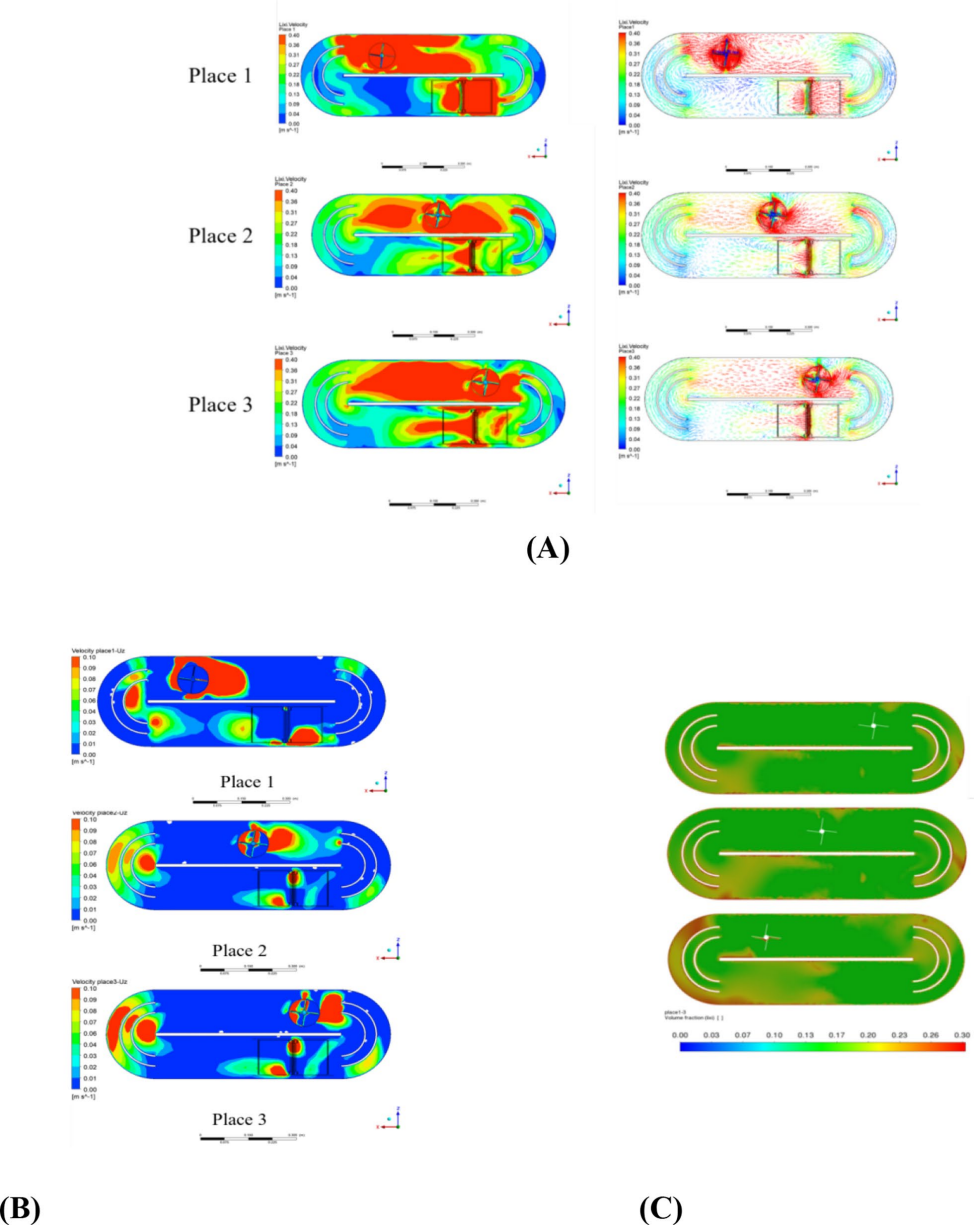
Schematic diagram of CFD numerical simulation results of automatic light-filling mixer running to different positions **A** Cloud diagram of flow distribution and vector diagram **B** Cloud diagram of average light gradient direction speed distribution **C** Cloud diagram of dead zone distribution position

Figure 4A presents the velocity contour and vector plots of the ORWP flow field under different stirring positions. At Place 1, the captured velocity data represent the original mixer position, showing an overall uneven flow velocity. The driving force of the main impeller has not fully encompassed the entire fluid region, resulting in incomplete medium circulation. In the initial stage, low-velocity zones are predominantly located on both sides of the curved sections and the anterior half of the A-EDS. The onset of stirring immediately elevated the flow velocity in the agitated area while eliminating low-velocity zones originally present near the baffle sides and walls. As the stirring device progressed to Place 2, fluid circulation throughout the flow domain was largely complete. Synergistically driven by stirring and A-EDS, the overall flow velocity was enhanced and stabilized, with only small low-velocity zones remaining. The average horizontal cross-sectional velocity reaches 0.24 m s^−1^, indicating a substantial improvement in the ORWP overall flow field. If the stirring reaches Place 3, the overall flow velocity further improves, with even fewer low-velocity zones. The long channel of the experimental raceway system was characterized by a high flow velocity and thorough mixing. From Place 1 to Place 3, the overall flow velocity improved, whereas the addition of A-EDS homogenized the velocity in formerly static regions across the flow field. This observation aligns with previous studies, which have demonstrated that optimized impeller design and placement can significantly enhance fluid dynamics in raceway ponds, reducing stagnant zones and improving mixing efficiency [21, 49]. Notably, our study advances this understanding by reducing dead zones (from 15.5 to 1.1%) and a 40% improvement in vertical mixing efficiency, exceeding the improvements reported in prior works.

Figure 4B shows the velocity plot along the optical gradient direction (Uz) and reveals that during the initial stage of the dynamic stirring system of the raceway pond, the highest Uz values were confined to the stirring region and partially extended to the adjacent A-EDS region. The average Uz was notably greater and affected a larger area than at the start of stirring, with the maximum Uz exceeding 0.1 m s^−1^ on the characteristic cross-section. After the ORWP internal fluid circulation reached equilibrium, higher Uz values were more prevalent in the A-EDS region, indicating intensified vertical fluid motion. The immense turbulent kinetic energy generated by stirring propels the maximum Uz on the characteristic cross-section above 0.1 m s^−1^. The presence of A-EDS facilitated the rapid conversion of horizontal fluid velocity into axial velocity, reinforcing vertical fluid motion. This finding is consistent with the work of Legrand et al. [24], who reported that optimized flow fields in raceway ponds can enhance vertical mixing, thereby improving light and nutrient distribution for microalgae growth. However, our study goes beyond previous research by quantifying the 14.4% increase in phycocyanin yield and the 43% improvement in carbon fixation rate, directly linking fluid dynamics optimization to enhanced biomass productivity and economic viability.

As illustrated in Fig. 4C, when the newly designed raceway pond system starts operating, incomplete fluid circulation restricts the influence of stirring-generated kinetic energy on a broader range of fluid elements. As the fluid navigated the curved sections, its kinetic energy was insufficient to mobilize and mix all the particles, leading to the formation of dead zones, primarily near the curved inner walls [42]. However, the extensive dead zones originally present on the baffle side were largely eliminated due to stirring. It reduced the overall dead zone ratio to 4.2% and led to a significant improvement over conventional raceway ponds. At Place 2, mixing was relatively thorough, and the stirring-induced kinetic energy influenced the entire flow field, with nearly complete fluid circulation. The incorporation of A-EDS further stabilized the optimized fluid motion and reduced dead zones on both sides of the curved sections, where only minor accumulations remained near the raceway pond walls. This indicated increased Uz and turbulent kinetic energy in these regions, which facilitated improved mixing of the algal suspension. As the stirring device advanced to Place 3, the internal flow field of the raceway pond significantly improved. Coupled with the flow guidance of A-EDS, dead zones were virtually eliminated as the dead zone ratio decreased to 1.1%, which demonstrated a remarkable improvement. Overall, the newly designed ORWP dynamic stirring system effectively minimized the dead zone ratio and alleviated particle accumulation at the pond bottom. This feature plays a crucial role in reducing microalgae sedimentation. The substantial reduction in the dead zone ratio from 15.5 to 1.1% underscored its efficacy.These results are supported by previous studies, which have shown that the integration of advanced mixing systems and flow guidance structures can significantly reduce dead zones and improve microalgae productivity [5, 33].

### Mixing time (MT), mass transfer coefficient (MTC) and light‒dark cycle

**Fig. 5:**
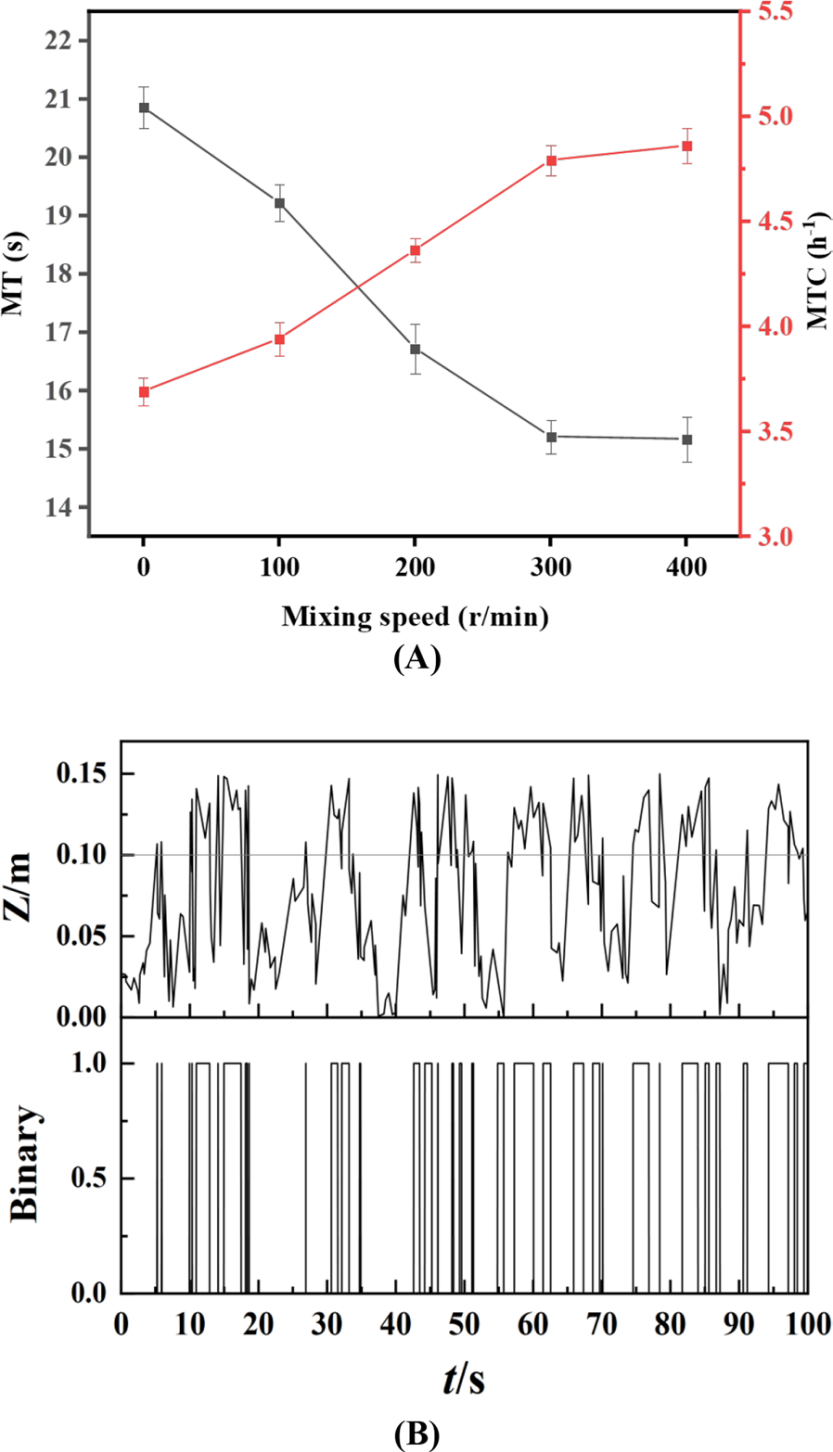
Changes in the mixing time, mass transfer coefficient and light‒dark cycle period during stirring. **A** Effects of stirring speed on changes in MT and MTC. **B** Trajectories of light-directed particles and binary diagrams of light‒dark cycle periods

Figure 5A illustrates the changes in MT and MTC in the novel ORWP with the primary impeller or paddle wheel speed maintained at 30 rpm. As the stirring speed increased from 0 to 400 rpm, the MT decreased from 20.9 ± 0.36 s to 15.2 ± 0.28 s, representing a reduction of nearly 37%, whereas the MTC increased from 3.7 to 4.9 h⁻^1^, indicating an efficiency improvement of approximately 32%. The stirring speed significantly impacts fluid mixing and mass transfer within the ORWP [16]. However, excessively high rotation speeds do not significantly increase mixing or mass transfer within a fixed volume of fluid [11]. When the stirring speed increased from 300 to 400 rpm, the improvement rates of MT and MTC were within 5%, suggesting no further significant enhancement, which is consistent with the previous CFD simulation results of the stirring zone.

The duration of vertical light gradient exposure plays a crucial role in microalgae growth, metabolism, product synthesis, and environmental adaptation [15]. After a certain cultivation period or when a specific concentration is reached, the ability of microalgae to utilize light at a depth of 5 cm below the water surface significantly decreases [19]. We therefore defined the uppermost 5 cm of the water column as the photic zone, while deeper areas were designated the aphotic zone. The highly turbulent kinetic energy generated by stirring notably increased the flow velocity of the medium along the light gradient, substantially increasing the amplitude and frequency of the particles' vertical motion [45].

Figure 5B depicts the particle trajectories over time in the dynamic stirring system of the ORWP and the reconstructed binary map of light‒dark cycles on the basis of the defined light and dark zones. Statistics revealed that 2.7 s passed through the light‒dark gradient for the filaments. Clearly, the illuminated area of the algal culture was no longer limited to a depth of 5 cm below the surface. Additionally, the increased proportion of the light zone allowed microalgae cells to remain longer in the illuminated region [6]. This demonstrated that the designed dynamic stirring device effectively enhanced the mixing and movement of microalgae cells in the vertical direction, ultimately facilitating improved microalgae production.

### Microalgae productivity

**Fig. 6:**
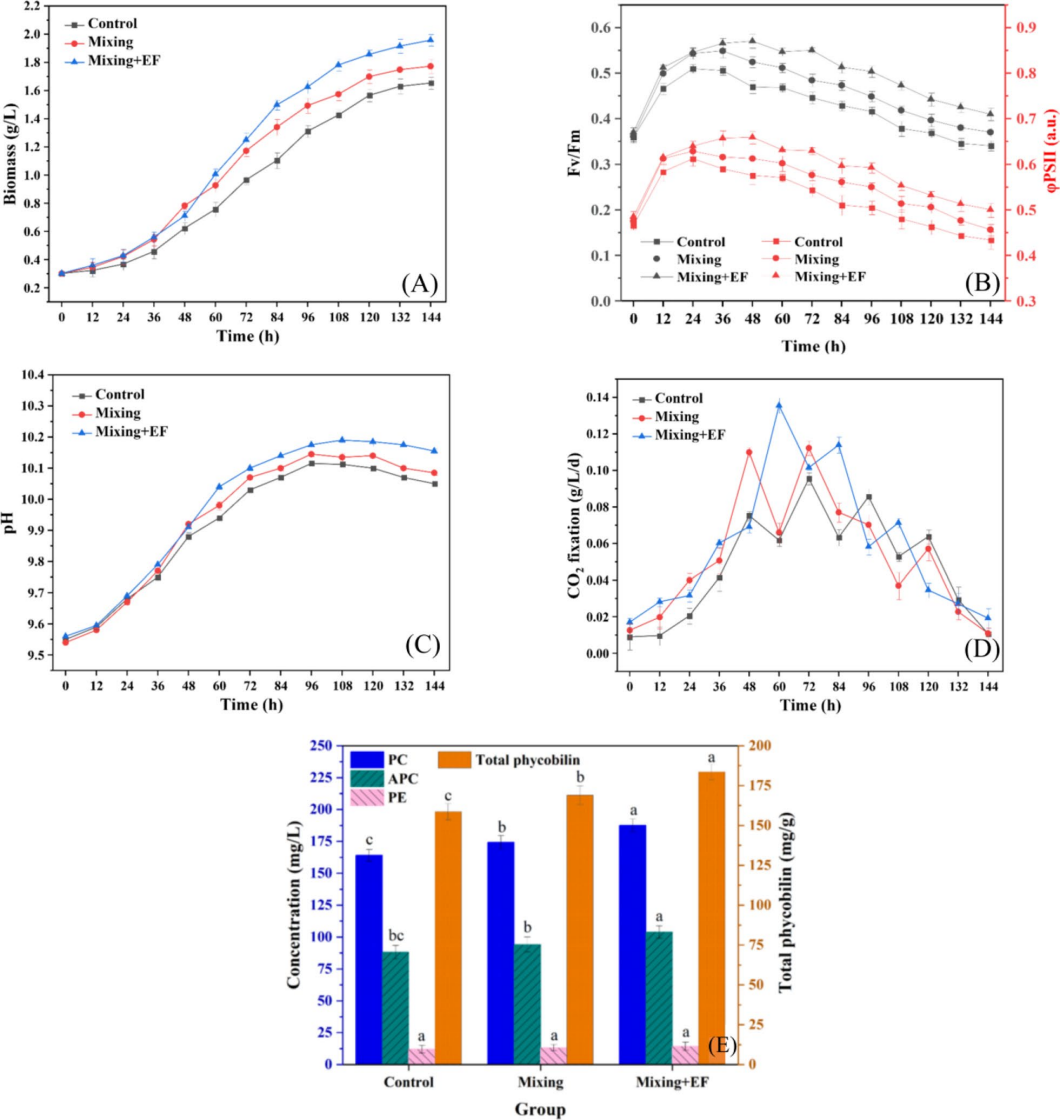
Changes in the content of various bioindicators of *L. fusiformis* in the optimized reactor under an electric field. **A** Dry mass. **B** Fv/Fm and ΦPSII. **C** pH. **D** CO_2_ fixation efficiency. **E** Changes of PC, APC, PE, and total phycobiliprotein content

As shown in Fig. 6A, all cultures required around 24 h to acclimate to the new environment, independent of the treatment (lag phase), followed by substantial growth over the next 72 h (log-phase). After 144 h of cultivation, the group with additional vertical mixing achieved a biomass yield of 1.77 g L⁻^1^, which was 7.2% higher than compared to the control. Without additional stirring, cells tend to accumulate at the bottom of the pond, leading to increased mortality [23]. Our approach significantly mitigates this issue. With the EF treatment, the biomass yield further increased, with that of the combined treatment of mixing + EF reaching 1.96 g L⁻^1^, which was approximately 18.8% greater than that of the control. The EF treatment likely affects the charge distribution and permeability of microalgal cell membranes, thereby improving their metabolic activities [12, 13], which is supported by previous findings showing that EF enhances the photosynthetic efficiency and growth rate of microalgae by increasing the fluidity of the cell membrane and facilitating the uptake of nutrients [8, 22]. For example, Choi et al. [8] demonstrated that applying a low-intensity EF to Chlorella vulgaris cultures doubled both the triacylglycerol content and biomass yield. Similarly, Kumar et al. [22] reported a 10% increase in biomass production of Tribonema minus during the log-phase under electrostatic stimulation. The bioprocessing advantages of EF stimulation are significant. EFs offer a non-chemical intervention that can enhance biomass and product synthesis while reducing the risk of contamination [12, 13]. Additionally, the EF approach is relatively simple to implement and can be integrated into existing raceway pond systems with minimal modifications. From a technical and economic perspective, EF stimulation has the potential to improve the overall efficiency and cost-effectiveness of microalgal cultivation systems, particularly for high-value products like phycocyanin.

The Fv/Fm values of all the experimental groups significantly increased within the first 24 h, indicating good acclimation to the experimental conditions (Fig. 6B). This was partly attributed to the low initial microalgae concentration in the ORWP, which allowed ample light to penetrate in all directions and reach the pond bottom [26]. In addition, rapid cell proliferation probably lowered the cellular PC-content, which usually interferes with the fluorescence signal [29]. With developing cultures, self-shading reduced light penetration, which caused low-light acclimation. In both vertical mixing and mixing + EF treatments, cells passlight and dark areas in shorter intervals, resulting in a flash effect [38], which enhanced photosynthetic activity. EF may also affect photosynthesis by impacting the electron transport chain or inducing new electron transport paths [41]. Compared with those of the control group, the Fv/Fm and ΦPSII values in the treatments were around 20%, and 15% higher.

Figure 6C shows that the pH changes in the algal cultures during production closely followed the increase in biomass. The mixing and mixing + EF groups presented higher pH values than did the control group, peaking at 10.2, which indicated greater productivity in these groups [14]. As productivity increases with increasing biomass, the capacity to utilize CO_2_ for photosynthesis also increases [20]. The dynamic turbulent vortex motion generated by the stirring devices not only increased the overall flow velocity of the algal culture but also improved contact between inorganic carbon and algal cells. Compared with the control group, the mixing and mixing + EF groups achieved maximum CO_2_ fixation efficiencies that were 17.9% and 43.2% greater, respectively (Fig. 6D). These findings demonstrated that microalgae utilized CO_2_ more efficiently for growth, resulting in greater nutrient consumption in the algal culture.

PC, a high-value light-harvesting pigment of cyanobacteria, such as *Limnospira fusiformis*, is widely used as a food supplement, in pharmacy, medicine, and biotechnological applications because of its antioxidant and anti-inflammatory properties [47]. The synergistic combination of optimized stirring and EF treatment significantly enhanced PC synthesis, with the PC content reaching 187.7 mg L⁻^1^ (productivity 31.28 mg·L⁻^1^ d⁻^1^) with the combined treatment (Fig. 6E). This value represents a 14.4% increase compared to the control (163.9 mg L⁻^1^) and exceeds reported values for “Arthrospira platensis” cultivated in conventional raceway ponds (27.1 mg L⁻^1^ d⁻^1^,[36]). The observed enhancement arises from two synergistic mechanisms: (1) increased light–dark cycling due to vertical mixing. The vertical stirring system reduced the light–dark cycle period to 2.7 s (Fig. 5B), promoting dynamic reorganization of phycobilisomes. Frequent alternations between light absorption (phycobilisome excitation) and dark recovery (energy transfer to reaction centers) minimized energy loss through non-photochemical quenching (NPQ), thereby increasing photosynthetic efficiency [45]. Additionally, this mixing device prevented photodamage in surface-near areas [33]. (2) EF-induced metabolic modulation with low-intensity EF (0.6 V·cm⁻^1^) probably impacts transmembrane potentials, increasing cell membrane permeability and facilitating nutrient uptake [8]. EF-induced mild oxidative stress could activate antioxidant defense pathways, triggering PC accumulation as a protective response [12, 13]. Importantly, while excessive light intensity can induce photoinhibition of Photosystem II (PSII) thereby indirectly suppressing PC synthesis by disrupting energy transfer within phycobilisomes [44]. Our dynamic light supplementation system maintained irradiance at 180 ± 5 μmol m⁻^2^ s⁻^1^, ensuring optimal conditions for both growth and pigment accumulation.

This study were able to effectively minimize inadequate mixing performance, susceptibility to deposition and dead zones, and low light supply during the production process. The introduced EF treatment method had additional positive effects on promoting microalgae growth and proved reliable in improving both the mixing and production capacity of microalgae in the ORWP as a whole.

## Conclusion

The performance of the raceway pond was enhanced by the innovative automated light-supplemented mixer. The reduction of dead zones promoted algae cell accumulation, improving mixing efficiency and light utilization. The light–dark cycle period of the cells was shortened to 2.7 s. The introduction of an electric field further facilitated microalgae growth. The combination of the automated light-supplemented mixer and electric field treatment led to an 18.8% increase in microalgae biomass, a 43% improvement in the maximum carbon fixation rate and the content of phycocyanin increased by 14.4%. The proposed novel automated light-supplemented mixer raceway pond system, combined with EF treatment, demonstrates significant productivity enhancements in laboratory-scale reactors. (depth/width ratio = 0.56). While our results validated the synergistic potential of combining hydrodynamic and electrochemical techniques, scaling-up to industrial raceway ponds with typical depth/width ratios < 0.1 requires further investigation of light penetration and energy efficiency trade-offs. Future research will focus on three key directions: (1) Pilot-scale validation with scaling up the system to 100–1,000 L raceway ponds with industrial geometries (depth/width ratio < 0.1) to evaluate performance under real-world conditions. (2) Energy optimization with developing solar-powered EF modules and low-shear impeller designs to reduce energy consumption. (3) Mechanistic studies: Employing transcriptomic (RNA-seq) and proteomic (LC–MS) approaches to elucidate the molecular mechanisms underlying EF-enhanced growth and phycocyanin synthesis.

## Data Availability

All data generated or analysed during this study are included in this published article and its supplementary information files.
